# Heart failure management insights from primary care physicians and allied health care providers in Southwestern Ontario

**DOI:** 10.1186/s12875-020-1080-y

**Published:** 2020-01-13

**Authors:** Narlon C. Boa Sorte Silva, Roseanne W. Pulford, Douglas S. Lee, Robert J. Petrella

**Affiliations:** 1 Centre for Studies in Family Medicine, Department of Family Medicine, Schulich School of Medicine and Dentistry, Western University, London, ON Canada; 2 School of Kinesiology, Faculty of Health Sciences, Western University, London, ON Canada; 3 ICES, Toronto, ON Canada; 4 Peter Munk Cardiac Centre of the University Health Network, Division of Cardiology, University of Toronto, Toronto, ON Canada; 5 Department of Family Practice, Faculty of Medicine, University of British Columbia, Vancouver, BC Canada; 6 Western Centre for Public Health and Family Medicine, Western University, 2nd Floor, 1465 Richmond St, London, ON N6G 2M1 Canada

**Keywords:** Heart failure management, Primary care, Health care professionals

## Abstract

**Background:**

It remains to be determined whether collaborative strategies to improve and sustain overall health in patients with heart failure (HF) are currently being adopted by health care professionals. We surveyed primary care physicians, nurses and allied health care professionals in Southwestern Ontario regarding how they currently manage HF patients and how they perceive limitations, barriers and challenges in achieving optimal management in these patients.

**Methods:**

We developed an online survey based on field expertise and a review of pertinent literature in HF management. We analyzed quantitative data collected via an online questionnaire powered by Qualtrics®. The survey included 87 items, including multiple choice and free text questions. We collected participant demographic and educational background, and information relating to general clinical practice and specific to HF management. The survey was 25 min long and was administered in October and November of 2018.

**Results:**

We included 118 health care professionals from network lists of affiliated physicians and clinics of the department of Family Medicine at Western University; 88.1% (*n* = 104) were physicians while 11.9% (*n* = 14) were identified as other health care professionals. Two-thirds of our respondents were females (*n* = 72) and nearly one-third were males (*n* = 38). The survey included mostly family physicians (*n* = 74) and family medicine residents (*n* = 25). Most respondents indicated co-managing their HF patients with other health care professionals, including cardiologists and internists. The vast majority of respondents reported preferring to manage their HF patients as part of a team rather than alone. As well, the majority respondents (*n* = 47) indicated being satisfied with the way they currently manage their HF patients; however, some indicated that practice set up and communication resources, followed by experience and education relating to HF guidelines, current drug therapy and medical management were important barriers to optimal management of HF patients.

**Conclusions:**

Most respondents indicated HF management was satisfactory, however, a minority did identify some areas for improvement (communication systems, work more collaborative as a team, education resources and access to specialists). Future research should consider these factors in developing strategies to enhance primary care involvement in co-management of HF patients, within collaborative and multidisciplinary systems of care.

## Background

Providing health care at a level of excellence to achieve and sustain improvements in patient health is the ultimate goal in clinical practice, this holds true particularly in patients diagnosed with heart failure (HF) [1]. Since HF is one of the main cardiovascular causes of death, it largely impacts the health care system, the patient’s family, caregivers, and physicians [2]. Moreover, with increasing survival rates following heart attacks, the incidence of people living with HF is rising in Canada, reaching approximately 50, 000 newly diagnosed each year according to the Heart and Stroke Foundation of Canada [3]. Therefore, implementing strategies to identify and counter-act limitations, barriers and challenges in HF management in primary care is a primordial step towards enhancing overall quality of health care services for patients.

Many factors have been identified to negatively influence the health status of HF patients, and one of these factors is the readmission of patients to acute care [4, 5]. It has been suggested that adoption of collaborative strategies among professionals (e.g., physicians, nurses, allied health and specialists) and institutions (hospitals and in-patient/out-patient clinics), as well as, implementation of post-discharge HF management programs, could result in lowering readmission rates in HF patients, and possibly lead to long-term, sustained health status [4, 6]. Furthermore, early collaborative care for HF patients, which could include collaboration between primary care physicians and specialist [2], seems to be particularly efficient in reducing mortality compared to primary care alone [2].

The current guidelines for the management of HF published by the Canadian Cardiovascular Society state that the management of these patients should be delivered within a system of care, following principles of chronic disease management and prevention [7]. However, it remains to be determined whether such strategies are currently being adopted by health care professionals. Therefore, the aim of this study was to investigate how health care professionals (e.g., family doctors, specialists, residents, nurses) from Southwestern Ontario manage HF patients, and identify the perceived limitations, barriers and challenges in achieving optimal HF management of patients in primary care.

## Methods

### Respondents

**Fig. 1:**
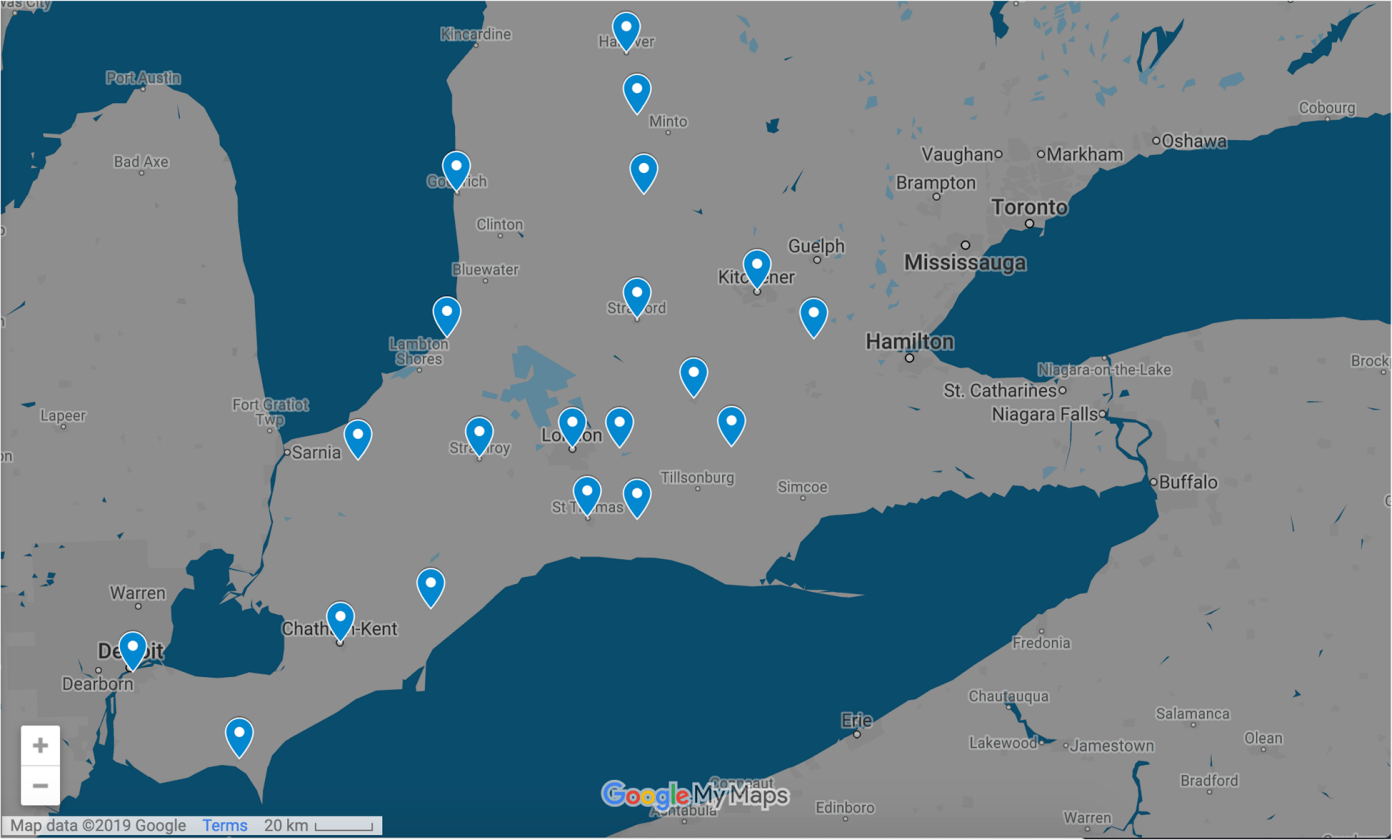
Respondent locations in the Heart Failure Management Survey across Southwestern Ontario. Note: The locations identified in map are Aylmer, Branchton, Chatham, Clifford, Dorchester, Goderich, Grand Bend, Hanover, Highgate, Kitchener, Leamington, Listowel, London, Norwich, St. Thomas, Stratford, Strathroy-Caradoc, Windsor, Woodstock, and Wyoming. The figure was derived from Map data©2019 Google

We recruited respondents from community-based practices and teaching hospitals across Southwestern Ontario. More specifically, respondents were located in 20 cities (based on their postal codes) bounded by the cities of Windsor and Essex County in the West, Hanover in the North, Kitchener to the East, and Leamington to the South (see Fig. 1). The online survey was sent to network listings of primary care and allied health professionals in practice or training from the Department of Family Medicine at Western University and included the following email networks: a) Clinical Academic Faculty and Adjunct Faculty in Family Medicine at Western University; b) Citywide Department of Family Medicine at Western University; c) Family Medicine Educational Research Networks at Western University; d) Family Medicine Residents at Western University; e) Amherstburg Family Health Team; f) North Perth Family Health Team; g) Stratford Family Health Network; and h) Peninsula Family Health Team. Email recipients were informed that their responses were anonymous and neither they themselves, their location nor their practice would be identified. In addition, recipients were informed that by answering the questionnaire they would be providing consent to participate.

### Survey development

Our team developed this survey based on field expertise and a review of pertinent literature in HF management, and one of the team members (RWP) underwent specific professional training prior to building the survey via the Institute for Healthcare Improvement (Open School), Quality Improvement [8]. The content of the survey was first created in a paper format and underwent critical revision by the study principal investigator (RJP), after which the questions were transformed to an online version using Qualtrics Software (2019 Qualtrics®, Provo, UT) in collaboration with Western University [9]. The survey included 87 items, including multiple choice and free text questions. All survey questions underwent pilot testing prior to release to participants. We collected participant demographic information and educational background, as well as information relating to general clinical practice and specific to HF management. The survey was approximately 25 min long and was administered in October and November of 2018.

### Data analysis

In this report, we analyzed only quantitative data collected via our online questionnaire. Qualitative responses from a sub-sample of respondents will be reported separately. All data generated by multiple choice questions were analyzed using descriptive statistics and no inferential statistical tests were performed on the data. We summarized the data as either frequency and percentage, or median and interquartile range (IQR). Although we removed outliers from the data during preprocessing stages in the statistical analysis, we explored the data to a maximum extent and no single answers were excluded. We conducted all analyses using IBM® SPSS® Statistics for Mac, Version 24.0 (Armonk, NY: IBM Corp.).

## Results

### Demographic information

This study included 118 health care professionals; 88.1% (*n* = 104) were physicians while 11.9% (*n* = 14) were identified as other health care professionals. Two-thirds of our respondents were females (*n* = 72) and nearly one-third were males (*n* = 38). Family physicians (*n* = 74) and family medicine residents (*n* = 25) composed the first and second largest group of doctors in this survey, respectively. As well, one general internist (group practice) and four specialists (emergency [*n* = 1], geriatric medicine [*n* = 1], palliative care [*n* = 1], sport and exercise medicine [*n* = 1]) were also surveyed. Among 14 other health care professionals, our sample included nurse practitioners, registered nurses, registered practical nurses, and others (registered dietitian and chronic disease team lead [*n* = 1], registered respiratory therapist [*n* = 1], and social worker [*n* = 1]). Please see Table 1 for a description of our respondents.

**Table 1:** Demographic information

Category	*N*	%^a^
Sex		
Female	72	61
Male	38	32.2
Other	3	2.5
Physicians		
Family Physicians	74	62.7
General Internist (group practice)	1	.8
Family Medicine Resident	25	21.2
Specialist	4	3.4
Other Health Care Professionals^b^		
Nurse Practitioner	6	5.1
Registered Nurse	3	2.5
Registered Practical Nurse	1	.8
Registered dietitian and chronic disease team lead	1	.8
Registered respiratory therapist	1	.8
Social worker	1	.8

^a^Percentage calculated from overall sample

^b^Please note data missing for one participant regarding their professional affiliation

### Educational background

The vast majority of our respondents completed their undergraduate medical training in Canada (*n* = 85), whereas others receive their training in other countries (*n* = 26), mostly outside North America (*n* = 24). As well, most respondents received their degree between the decades of 2000 to 2009 (*n* = 27) and 2010 to 2019, (*n* = 44). Moreover, more than two-thirds of respondents indicated receiving post-graduate medical training (*n* = 78), mostly in family medicine (*n* = 55), and mostly in Canada (*n* = 74) while 70 received Certification by the College of Family Physicians of Canada (CCFP). Please see Table 2 for detailed information.

**Table 2:** Educational background and current practice

Category	*N*	%^a^
Education		
Country of graduation		
Canada	85	72
USA	2	1.7
Other Counties	24	20.3
Year of graduation		
1970 to 1979	8	7.3
1980 to 1989	14	12.7
1990 to 1999	17	15.5
2000 to 2019	27	24.5
2010 to 2019	44	40.0
Post-graduate Medical Training^b^		
Family Medicine	55	46.2
Other	29	24.3
No Post-graduate Medical Training	43	36.1
Practice Location		
Rural	36	30.5
Urban	78	66.1
Hospital Privileges		
No	38	32.2
Yes	76	64.4
Type of Hospital Setting		
Academic Health Sciences Centre (AHSC)	41	34.7
Community hospital	28	23.7
Emergency department (in community hospital or AHSC)	2	1.7
Non-AHSC teaching hospital	2	1.7
Other hospital	1	.8

^a^Percentage calculated from overall sample

^b^Eight respondents reported having other post-graduate medical training in addition to family medicine

### General clinical practice

More than two-thirds of our respondents reported having hospital privileges (*n* = 76). Of those with hospital privileges, the vast majority reported having access to an Academic Health Sciences Centre (*n* = 41) or community hospital (*n* = 28). Five respondents indicated they practiced in solo practice, four of which indicated having a nurse available. With regards to their main practice location, one-third of respondents indicated practicing mainly in rural (*n* = 36), while the majority reported practicing in urban locations (*n* = 78). Please see Table 2 for detailed location information. In addition, survey respondents indicated seeing on average 70 patients (median, IQR = 50) on a weekly basis, with some respondents seeing as many as 220 patients per week. For urgent matters, our respondents (*n* = 83) reported that most patients would have a first available appointment for the same day; similarly, for non-urgent matters, respondents (*n* = 66) reported that patients are seen within the same week. Please see Table 3 for details.

**Table 3:** Patient visits

Category	*N*	%^a^
General Patient Visits		
Urgent matter		
Same day	83	70.3
First available, but not same day	10	8.5
Other	9	7.6
Unsure	3	2.5
Non-urgent matter		
Same week	66	55.9
When available, but not same week	23	19.5
Other	9	7.6
Unsure	6	5.1

^a^Percentage calculated from overall sample

**Fig. 2:**
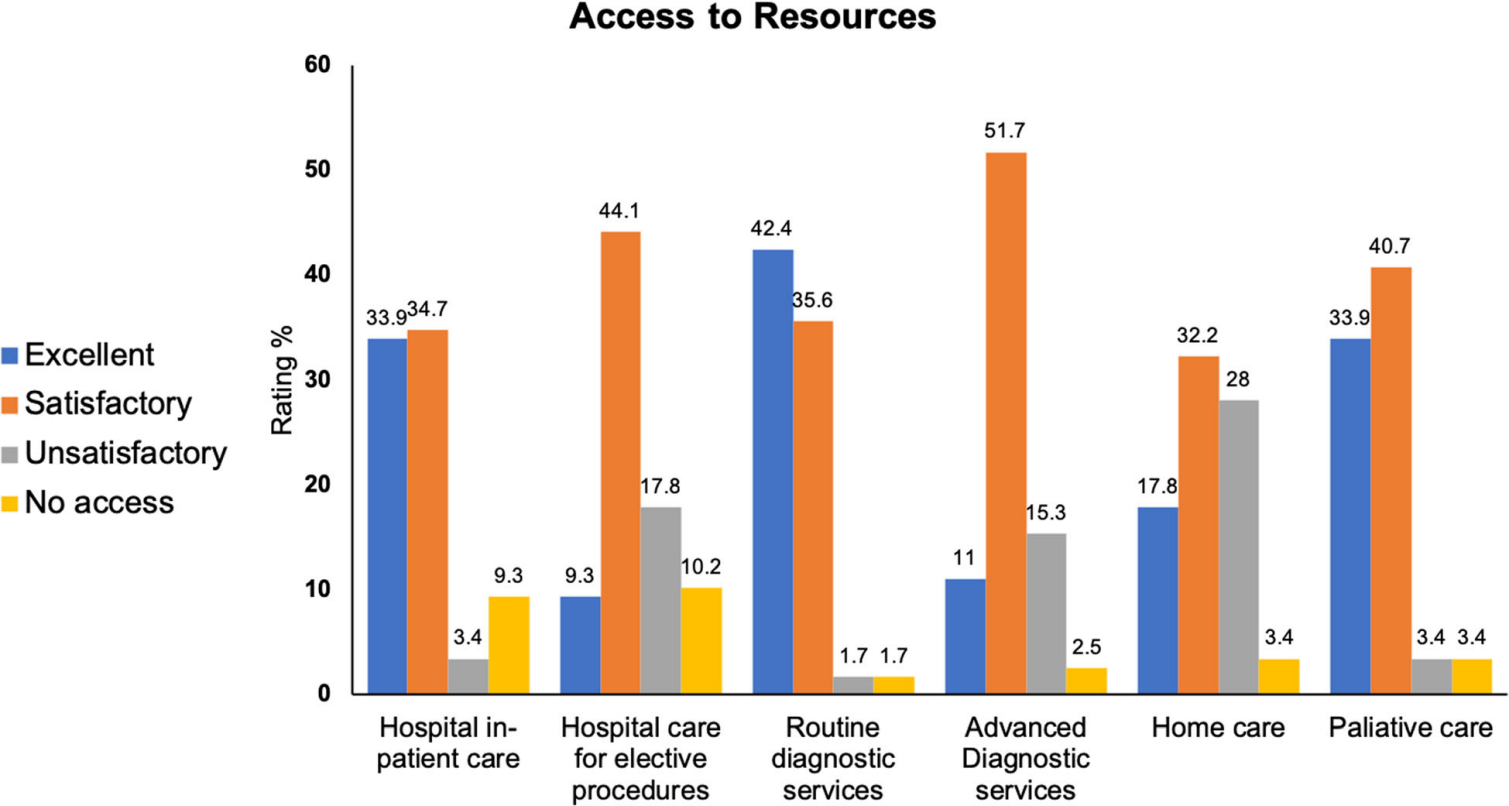
Participant ratings of access to resources in general clinical practice. Note: data presented as percentage of overall sample

We requested respondents to rate their access to different resources for treating HF patients; these are reported in Fig. 2. Briefly, most respondents reported satisfactory to excellent access to hospital in-patient care on an urgent basis, hospital care for elective procedures, and routine diagnosis services; however, there were more unsatisfactory to satisfactory ratings of access to advanced diagnosis services (e.g., magnetic resonance imaging, computerized tomography), home care, and palliative care.

### Collaboration in general clinical practice

Regarding collaboration in general clinical practice, our respondents were asked whether they participated in an inter-professional collaborative practice, excluding the hospital environment and referrals. Nearly two-thirds of respondents (*n* = 67) indicated that they have inter-professional collaborative practice. Among the providers involved in collaborative teams, respondents were given a list of specialists (e.g., cardiologists, general internists, other physicians, psychiatrists etc.) and were asked to select the ones with whom they mostly collaborate. Our results indicate that other health care providers (*n* = 56, e.g., nurse practitioners, physiotherapists, and occupational therapists), other physicians (*n* = 32 e.g., family physicians, emergency medicine, geriatrics, palliative care, urologists) and psychiatrists (*n* = 23) were the most common professionals involved in the inter-profession collaborative practice, followed by general internal medicine (*n* = 11), cardiologists (*n* = 9), obstetricians/gynecologists (*n* = 8), orthopedic surgeons (*n* = 5), general surgeons (*n* = 4) and dermatologists (*n* = 2).

### Heart failure management

**Fig. 3:**
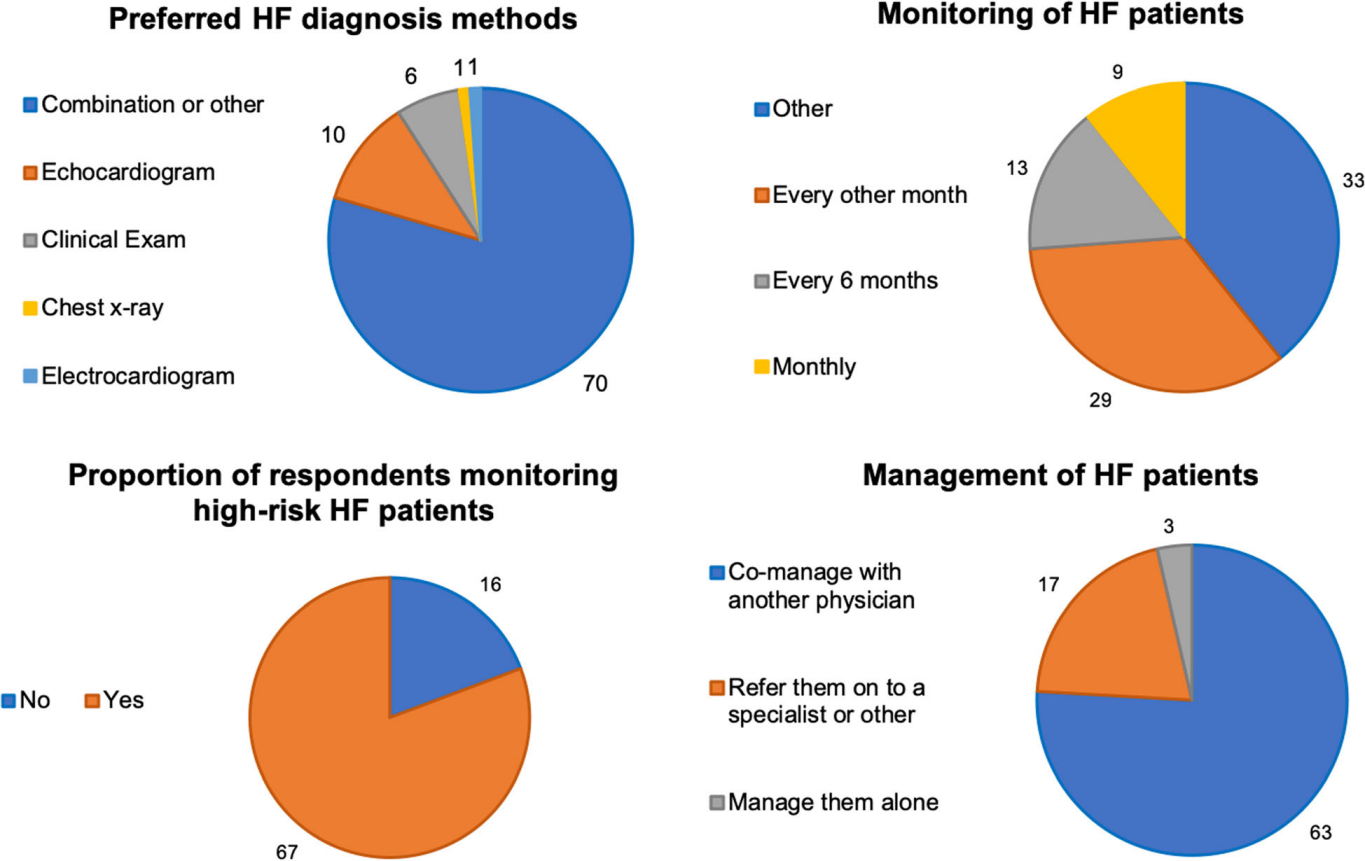
Heart failure diagnosis, monitoring and management data. Note: data presented as counts (n). HF = heart failure

Regarding HF patients, our respondents indicated seeing on average 20 patients (IQR = 34) per week. When asked about means to identify/diagnose HF patients in their practice, most respondents reported using a combination of methods/techniques (*n* = 70, e.g., combination of electrocardiogram, echocardiogram, and chest x-ray), while others reported using solely echocardiogram (*n* = 10), please see Fig. 3 for details. Additionally, other respondents also indicated using clinical exam and medical history in their diagnostic process. Furthermore, one-quarter of respondents (*n* = 29) indicated monitoring their HF patients every 6 months; however, the majority of respondents (*n* = 33) reported doing so in a timeline other than provided in the survey. This second group indicated seeing patients approximately every 3 months on average (*n* = 18), and that monitoring would be heavily influenced by the patient’s medical condition (e.g., 15 respondents indicated that number and frequency of visits would increase with worsening of patient’s health). Accordingly, most respondents reported (*n* = 67) having high-risk patients in their practice, with an estimated number of high-risk patients ranging from 1 to 50 per practice.

Respondents in our study also reported co-managing their HF patients with another physician (*n* = 63), while some would rather refer these patients on to a specialist or others (*n* = 17; others included respiratory therapist and certified educator for HF education program), with a minority of physicians preferring to manage patients alone (*n* = 3). Respondents’ choices for co-managing patients with other physicians are shown in as show in Table 4, stratified by respondent’s health care professional category (e.g., family physician, resident etc.). When asked whether they managed HF patients differently from patients with other chronic health conditions (e.g., diabetes), most responded (*n* = 50) no differences in management, while other respondents (*n* = 33) indicated managing differently. Further, when probed regarding how respondents manage HF patients compared to other chronic health conditions, the majority of survey respondents indicated that their HF patients would need more co-management, more office visits, and more urgent visits.

**Table 4:** Heart failure management

Category^a^	Family Physician	Resident	Specialist	Internist	Other HCP	Total
HF Management	**60 (50.8)**	**13 (11)**	**2 (1.7)**	**1 (0.8)**	**7 (5.9)**	**83 (70.3)**
Co-manage with another physician	47 (39.8)	8 (6.8)	2 (1.7)	1 (0.8)	5 (4.2)	63 (53.4)
Refer patients to specialist or other	10 (8.5)	5 (4.2)	–	–	2 (1.7)	17 (14.4)
Manage patients alone	3 (2.5)	–	–	–	–	3 (2.5)
HF Co-Management^b^						
Cardiologist *alone*	23 (19.5)	2 (1.7)	1 (0.8)	–	–	26 (22)
Internist *alone*	8 (6.7)	1 (0.8)	–	–	2 (1.7)	11 (9.3)
Other Family Physician *alone*	1 (0.8)	1 (0.8)	–	1 (0.8)	1 (0.8)	4 (3.4)
Cardiologist *and* Internist	13 (11)	2 (1.7)	–	–	–	15 (12.7)
Cardiologist *and* other Family Physician	1 (0.8)	1 (0.8)	–	–	1 (0.8)	3 (2.5)
Cardiologist, Internist *and* other Family Physician	1 (0.8)	–	–	–	1 (0.8)	2 (1.7)
Others^c^	–	1 (0.8)	1 (0.8)	–	–	2 (1.7)

Bold numbers indicate total number of respondents per HCP category

*HCP* Health care professionals, *HF* heart failure

^a^Data reported as number and percentage derived from all initial survey respondents (*n* = 118)

^b^For respondents who reported co-managing patients with another physician

^c^A participant reported co-managing with a nephrologist, while other with nurse practitioner

As well, the extreme majority of individuals (*n* = 80) in this survey suggested they would like to manage their HF patients as part of a team, particularly in co-management (*n* = 78); and 47 respondents reported that they are currently satisfied with the way they management HF patients (family physicians = 32; residents = 8; specialist = 1; and other health care providers = 6); others (*n* = 36) suggested that there are limitations preventing them from managing patients the way they would like. For these latter respondents, we further requested they specify these possible limitations, results are shown in Table 5, stratified by respondent’s health care professional category (e.g., family physician, resident etc.). Most respondents indicated that they would like to have access to more resources (*n* = 27), followed by more experience (*n* = 15) and lastly more education (*n* = 12) to manage HF patients in their practice. Finally, we inquired how respondents utilized the Ministry of Health and Long-Term Care (MOHLTC) Heart Failure Management Incentive fee code Q050A. Only 26.3% reported using the incentive (*n* = 31), while 29.7% indicated that they do not use it (*n* = 35), and 12.7% reported not being aware of the incentive (*n* = 15).

**Table 5:** Limitations preventing management of heart failure patients

Category^a^	Family Physician	Resident	Specialist	Internist	Other HCP	Total
Lacking Experience	**11 (9.3)**	**3 (2.5)**	**–**	**–**	**1 (0.8)**	**15 (12.7)**
HF Guidelines	9 (7.6)	1 (0.8)	–	–	1 (0.8)	11 (9.3)
Current drug therapy	9 (7.6)	1 (0.8)	–	–	1 (0.8)	11 (9.3)
Medication management for patients with HF and co-morbidities	8 (6.8)	2 (1.7)	–	–	1 (0.8)	11 (9.3)
How to read test results	5 (4.2)	1 (0.8)	–	–	1 (0.8)	7 (5.9)
Patient/family/caregiver education	6 (5.1)	1 (0.8)	–	–	1 (0.8)	8 (6.8)
No limitations in experience category	17 (14.4)	2 (1.7)	1 (0.8)	1 (0.8)	–	21 (17.8)
Lacking Education/Training	**9 (7.6)**	**3 (2.5)**	**–**	**–**	1 (0.8)	**13 (11)**
HF Guidelines	8 (6.8)	1 (0.8)	–	–	–	9 (7.6)
Current drug therapy	7 (5.9)	1 (0.8)	–	–	–	8 (6.8)
Medication management for patients with HF and co-morbidities	6 (5.1)	1 (0.8)	–	–	1 (0.8)	8 (6.8)
How to read test results	5 (4.2)	1 (0.8)	–	–	1 (0.8)	7 (5.9)
Patient/family/caregiver education	5 (4.2)	–	–	–	–	5 (4.2)
No limitations in education/training category	19 (16.1)	2 (1.7)	1 (0.8)	1 (0.8)	–	23 (19.5)
Lacking Resources	**20 (16.9)**	**5 (4.2)**	**–**	**1 (0.8)**	**1 (0.8)**	**27 (22.9)**
Practice set up (i.e., availability of nurses and support staff)	15 (12.7)	4 (3.4)	–	1 (0.8)	1 (0.8)	21 (17.8)
Communication resources (i.e., other doctors, specialists)	12 (10.2)	2 (1.7)	–	1 (0.8)	1 (0.8)	16 (13.6)
Time	11 (9.3)	3 (2.5)	–	–	–	14 (11.9)
Patient load	4 (3.4)	–	–	1 (0.8)	–	5 (4.2)
Money	1 (0.8)	1 (0.8)	–	–	–	2 (1.7)
No limitations in resources category	8 (6.8)	–	1 (0.8)	–	–	9 (7.6)
Other	**11 (9.3)**	**–**	**1 (0.8)**	**–**	**–**	**12 (10.2)**

Bold numbers indicate total number of respondents per HCP category

*HCP* Health care professionals, *HF* heart failure

^a^Data reported as number and percentage derived from all initial survey respondents (*n* = 118)

## Discussion

### Main findings

The current challenges to provide optimum care for patients with HF affect not only the patients themselves, but extends to their families, caregivers, physicians and other health care professionals. With a growing number of newly diagnosed cases every year, the burden on all the sectors of the health care sector and society is inescapable. Even though current recommendations stipulate a multidisciplinary approach to HF management and care [7], it remains to be determined whether such strategies are currently taking place in primary care. For example, given the low utilization of a special HF fee code we observed, this would suggest that introducing such supportive funding investments require supportive Continuing Medical Education (CME) programming. The overall goal of this study was to assess how health care professionals manage HF in their clinical practice in Southwestern Ontario, as well as, identify any perceived limitations, barriers and challenges in managing these patients.

We report that most of our respondents indicated participating in a collaborative practice setting, particularly with other health care professionals such as nurses and physiotherapists, as well as other family physicians. Similarly, despite nearly a third of respondents not having hospital privileges and practicing in rural areas, both of which could limit inter-professional collaboration, most respondents indicated co-managing their HF patients with other health care professionals, including cardiologist and internists. Co-management seems to be greatly valued in this context, since the vast majority of respondents reported preferring to manage their HF patients as part of a team rather than alone. This is an important pillar/component of the chronic care model (CCM) proposed by Wagner and colleagues [10, 11]. The development of collaborative and productive relationships between health care professionals is emphasized in this model. The model pertains to multidimensional approach to care, strengthening the relationships between health care providers, patients and their family, as well as integrating public and private resources in the community to aid in the daily management of chronic conditions. This model was not fully explored here but could benefit from future research in the management of HF patients.

Most of our respondents indicated being satisfied with the way they currently manage their HF patients, however, others indicated otherwise. The main limitation affecting this issue seemed to be related to practice set up (i.e., availability of nurses and support staff) and communication resources (i.e., with other doctors and specialists), followed by experience and education relating to HF guidelines, current drug therapy and medical management. Although we did not perform any inferential statistical test for this particular report, it is possible to consider that some of these factors might well be associated and even influenced by other factors, such as practice location (rural versus urban) especially in regards to greater or easier access to resources. Moreover, academic background, year of graduation, presence (or not) of a post-graduation degree, and hospital privileges might play important roles in suboptimal levels of satisfaction relating to how respondents in our survey manage their HF patients. One could consider that investing in CME specifically in HF management, would aid in addressing some of the main limitations indicated by our survey respondents. This could also aid in higher utilization of the HF fee code, as mentioned earlier. These observations, however, remain only as speculative.

### Limitations

Our survey was designed based on previous knowledge gathered from literature review and was critically appraised by experts in the field, however, it is not without limitations. One of the main constraints of the online survey is that we could not ensure that all respondents completed the survey thoroughly, with only two-thirds of respondents completing all questions in the questionnaire (*n* = 80); however, 96 (81%) respondents completed at least half of the survey. This does not particularly invalidate the data collected; however, it could bias our results by reflecting only the answers of those who completed the questionnaire. We also report results from a diverse group including individuals with different needs (e.g., senior doctors vs residents). In addition, although we attempted to design a relatively short survey, we appreciate many respondents may have not completed the survey due to time constraint. Further, due to a small sample size, we analyzed our data summarizing information across all different individuals who took the survey, however the majority of the respondents were either family physicians or family medicine residents; therefore, our results may not generalize outside the context of our sample in this study. As well, our sample of respondents were from Southwestern Ontario only and may not reflect practice and challenges in other parts of the province, particularly because the prevalence of heart disease in Southwestern Ontario is higher than in other parts of the province [12], even though distribution of physicians is similar across regions, as well as the prevalence of other chronic conditions such as diabetes and chronic obstructive pulmonary disease [12, 13]. Finally, due to the nature of the data collected, we are not able to perform hypothesis testing to confirm our speculations.

## Conclusions

Health care professionals involved in HF management, including family physicians, specialists, nurses, allied health professionals and family medicine residents were surveyed in this study. Our main findings indicate that these professionals are involved in collaborative HF management and prefer to co-manage their patients in multidisciplinary teams. Most respondents reported being satisfied with the current way in which they manage their HF patients. For those who reported being currently unsatisfied, the main factors that could be considered barriers and/or limitations were to access to resources, especially practice set up and communication with other health care professionals, as well as limitations with education and experience in regard to HF guidelines, current drug therapy and medical management. Future research should consider these factors in developing strategies to enhance primary care for HF patients especially with regards to managing these patients in collaborative and multidisciplinary system of care. The identification of and characterisation of subgroups facing these barriers and/or limitations in their practice would facilitate targeted intervention aiming at counteracting these items.

## Data Availability

The datasets used and/or analysed during the current study are available from the corresponding author upon request.

## Abbreviations

HF: Heart failure
IQR: Interquartile range
MOHLTC: Ministry of Health and Long-Term Care
